# CD28 is superior to 4-1BB costimulation in generating CAR-NK cells for tumor immunotherapy

**DOI:** 10.1186/s40164-025-00618-7

**Published:** 2025-03-03

**Authors:** Pengchao Zhang, Xuejia Feng, Xiangyun Niu, Zhongming Liu, Minghui Li, Maoxuan Liu, Dehong Yan, Guizhong Zhang, Xiaochun Wan

**Affiliations:** 1 Center for Protein and Cell-based Drugs, Institute of Biomedicine and Biotechnology, Shenzhen Institutes of Advanced Technology, Chinese Academy of Sciences, Shenzhen, 518055 People’s Republic of China; 2 University of Chinese Academy of Sciences, Beijing, 100049 PR China

**Keywords:** CAR-NK, Transcriptomics, CD28, 4-1BB, MAP3K8

## Abstract

Chimeric antigen receptor (CAR)-NK therapy holds great potential for tumor treatment, but current CAR designs are primarily optimized for T cells, raising concerns about their suitability for NK cells. This study compared two dominant CAR designs used in T cells—CD28-CD3ζ (28z) and 4-1BB-CD3ζ (BBz)—and found that CD28 costimulation offers superior functionality in NK cells. 28z CAR-NK cells exhibited significantly better activation, cytotoxicity, and in vivo anti-tumor efficacy than BBz CAR-NK cells, with similar persistence and tumor infiltration. 28z CAR more effectively recruited the ZAP70 kinase and upregulated multiple key factors involved in immune activation, potentially augmenting CAR-NK cell function. MAP3K8, a kinase involved in inflammation and the MAPK signaling pathway, was identified as a critical mediator in enhancing 28z CAR-NK cell function. Silencing or inhibiting MAP3K8 impaired the anti-tumor activity of 28z CAR-NK cells, while its overexpression substantially improved the function of BBz CAR-NK cells. These findings provide new insights into how CD28 costimulation boosts CAR-NK cell efficacy, supporting its use into NK cell-specific CARs for cancer immunotherapy, and highlight MAP3K8 as a potential target for optimizing BBz CAR-NK cell therapy.

**Supplementary Information:**

The online version contains supplementary material available at 10.1186/s40164-025-00618-7.

To the Editor,

Chimeric antigen receptor (CAR)-NK cells have demonstrated strong anti-tumor potential and safety in preclinical and clinical trials, garnering increasing attention [1]. Most CARs used in NK cells are predominantly designed for T cells, but it remains uncertain if these T cell-centric CARs are suitable for NK cells. To fully realize CAR-NK cells’ clinical potential, it is crucial to evaluate these CARs, particularly costimulatory domains, in NK cells to identify more effective designs or develop new CARs tailored for NK cells.

A recent study indicates that CD28, unlike 4-1BB, can synergize with CD3ζ to recruit key immune activation kinases like ZAP70 and LCK more effectively, enhancing CAR-NK cell function [2]. This highlights CD28’s superiority over 4-1BB in CAR signaling for NK cells. However, downstream molecular pathways, particularly transcriptomic changes, were not fully explored. Our study confirms CD28’s advantages and, through RNA-Seq and functional experiments, reveals how CD28 costimulation rewires activation networks in CAR-NK cells. We also identified MAP3K8, a key regulator of the MEK1/2-ERK1/2 pathway [3, 4], as crucial for enhancing CAR-NK cell function. Our findings emphasize CD28’s suitability in CAR-NK cells and provide insights for optimizing CAR-NK therapies.

## CD28 CAR-NK cells exhibit superior anti-tumor function than 4-1BB CAR-NK cells

**Fig. 1:**
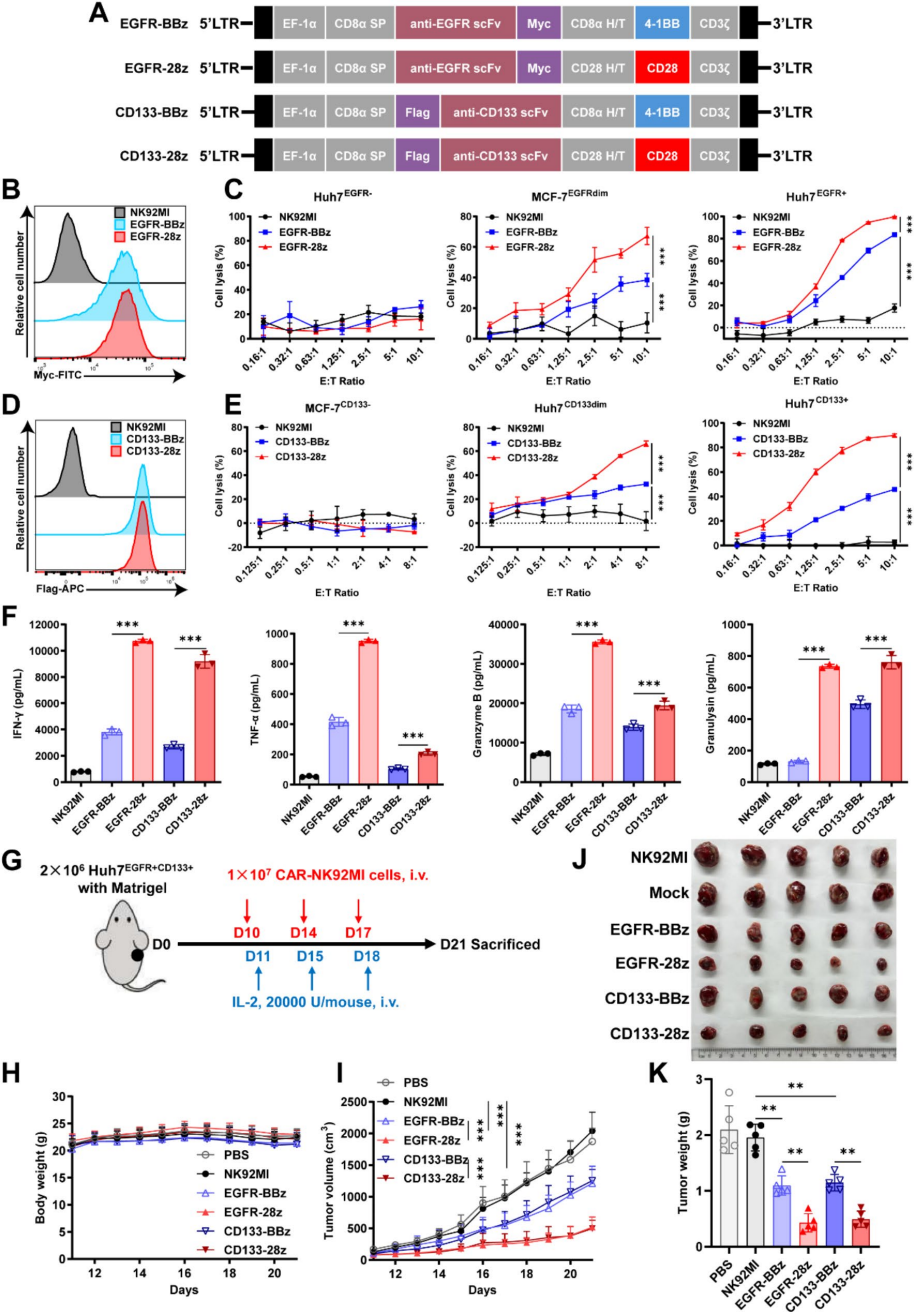
Functional comparison between 28z CAR-NK and BBz CAR-NK cells. (**A**) Structure schematic diagram of the 4-1BB or CD28 costimulatory domain-based CARs that target EGFR or CD133, named EGFR-BBz, EGFR-28z, CD133-BBz, and CD133-28z, separately. SP, signal peptide. H/T, hinge-transmembrane domain. (**B** and **D**) Flow cytometry analysis of EGFR (**B**) and CD133 (**D**)-targeting CARs expression on the surface of NK92MI. (**C** and **E**) Cytotoxicities of EGFR (**C**) and CD133 (**E**)-targeting CAR-NK92MI to antigen-negative (left), low expression (middle), and positive (right) target cells. (**F**) CAR-NK92MI cells were cocultured with Huh7^EGFR + CD133+^ cells at an E: T ratio of 2:1 for 24 h, then the secreted IFN-γ, TNF-α, Granzyme B, and Granulysin were measured by cytometric bead array (CBA). (**G**) Diagram of experimental design of Huh7 xenograft model. M-NSG mice were subcutaneously injected with 2 × 10^6^ Huh7 cells with Matrigel (day 0). Once the tumor volume reached nearly 100 mm^3^ (day 10), mice were randomly grouped and injected (i.v.) with 1 × 10^7^ CAR-NK92MI cells. 20,000 U/mouse recombinant human IL-2 was injected (i.v.) one day after the CAR-NK92MI infusion. *n* = 5 mice per group. Body weight (**H**) and tumor volume (**I**) were monitored daily. Tumors (**J**) were dissected from the mice at the end point and the tumor weights (**K**) were recorded. All results are presented as mean ± SD. The differences were analyzed by one-way or two-way ANOVA analysis. *p < 0.05; **p < 0.01; ***p < 0.001; n.s., not significant

To compare CD28 and 4-1BB costimulatory signals in NK cells, we engineered second-generation CARs targeting EGFR with either CD28 or 4-1BB costimulatory domains (Fig. 1A). These were expressed in NK92MI and YTS NK cells, termed 28z CAR-NK and BBz CAR-NK, respectively (Fig. 1B, S1A). In vitro assays showed that 28z CAR-NK cells had stronger tumor-killing activity than BBz CAR-NK cells (Fig. 1C, S1B), regardless of target antigen levels (Fig. S1C). This enhanced cytotoxicity was consistent across different targets, including CD133 (Fig. 1D-E, S1D-E), and in both hematologic and solid tumor cells (Fig. S2). Similarly, 28z CAR-NK cells secreted higher levels of cytokines (IFN-γ and TNF-α) and degranulation molecules (granzyme B and granulysin) (Fig. 1F). In vivo studies confirmed that 28z CAR-NK cells displayed superior antitumor activity than BBz CAR-NK cells (Fig. 1G-K), driven by differences in activation and cytotoxicity, rather than persistence or tumor infiltration (Fig. S3). These findings highlight that 28z CARs outperform BBz CARs in NK cells.

## CD28 costimulation enhance NK cell function by transcriptomic alteration

**Fig. 2:**
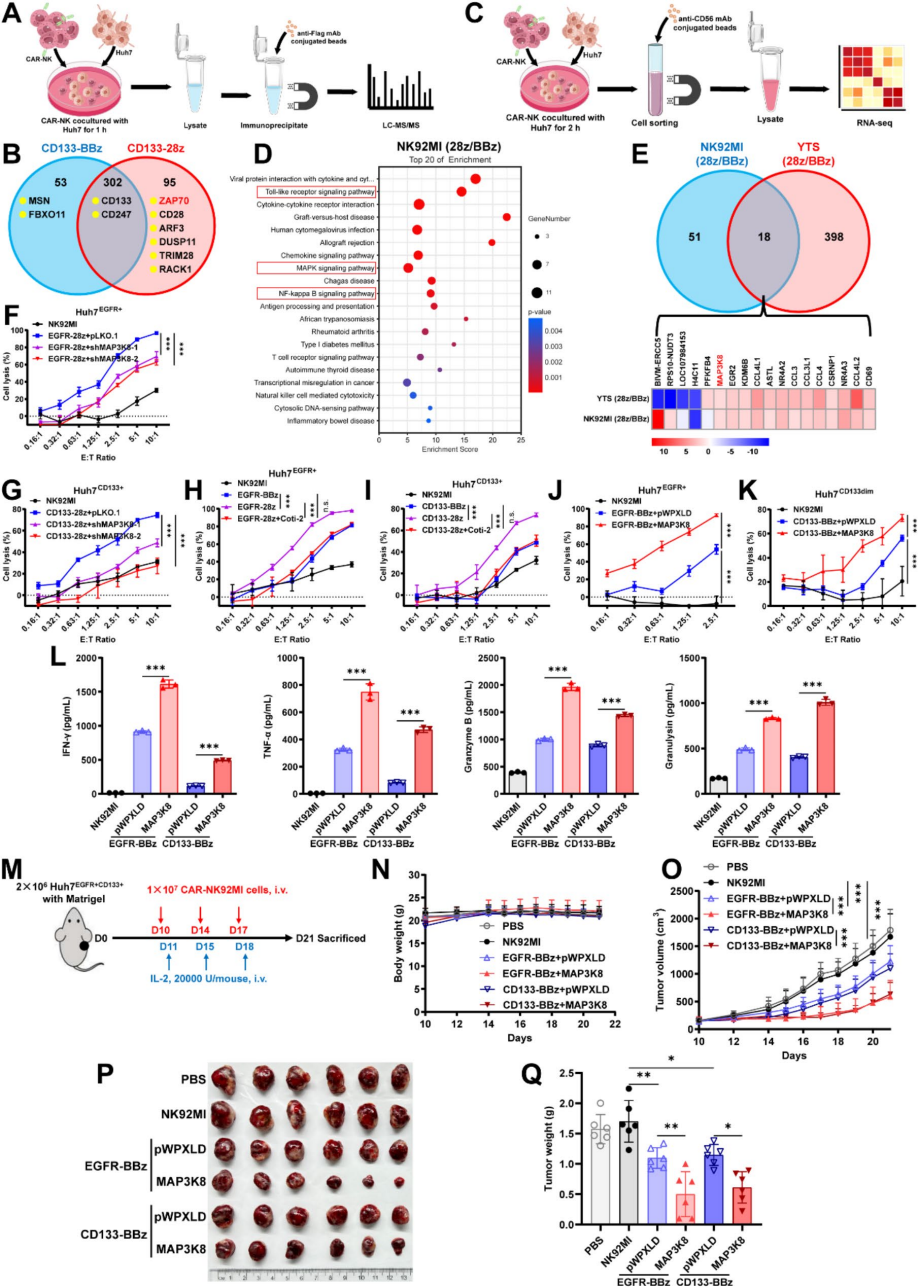
The transcriptomic mechanism that 28z CAR-NK outperform BBz CAR-NK cells. (**A**) Schematic diagram of BBz and 28z CAR interactome analysis by Co-IP coupled with LC-MS/MS assay. (**B**) Venn diagram of the interacting proteins of BBz and 28z CAR. (**C**) Schematic diagram of RNA sequencing analysis of BBz and 28z CAR-NK. (**D**) KEGG pathway enrichment bubble chart of 28z CAR-NK92MI versus BBz CAR-NK92MI. (**E**) Differential genes Venn diagram of 28z versus BBz in CAR-NK92MI and CAR-YTS cells. (**F-I**) Effect of MAP3K8 knockdown (**F, G**) and inhibition (**H, I**) on cytotoxicity of 28z CAR-NK92MI to Huh7^EGFR + CD133+^ cells. For MAP3K8 inhibition, 28z CAR-NK92MI be pretreated with 2.5 µM MAP3K8 inhibitor (Coti-2) for 1 h, then cocultured with target cells for 6 h in the presence of 2.5 µM Coti-2. (**J** and **K**) Effect of MAP3K8 overexpression on cytotoxicity of BBz CAR-NK92MI to Huh7^EGFR + CD133+^ cells. (**L**) Untransduced NK92MI, BBz CAR-NK92MI, and MAP3K8-overexpressed BBz CAR-NK92MI cells were cocultured with Huh7^EGFR + CD133+^ cells at an E: T ratio of 2:1 for 24 h, then the secreted IFN-γ, TNF-α, Granzyme B, and Granulysin were measured by CBA. (**M**) Diagram of experimental design of Huh7 xenograft model. M-NSG mice were subcutaneously injected with 2 × 10^6^ Huh7 cells with Matrigel (day 0). Once the tumor volume reached nearly 100 mm^3^ (day 10), mice were randomly grouped and injected (i.v.) with 1 × 10^7^ CAR-NK92MI cells. 20,000 U/mouse recombinant human IL-2 was injected (i.v.) one day after the CAR-NK92MI infusion. *n* = 6 mice per group. Body weight (**N**) and tumor volume (**O**) were monitored daily. Tumors (**P**) were dissected from the mice at the end point and the tumor weights (**Q**) were recorded. All results are presented as mean ± SD. The differences were analyzed by one-way or two-way ANOVA analysis. *p < 0.05; **p < 0.01; ***p < 0.001; n.s., not significant

Using immunoprecipitation (IP) combined with liquid chromatography-tandem mass spectrometry (LC-MS/MS), we found that 28z CAR more efficiently recruited ZAP70 (Fig. 2A-B, S4A), a kinase critical for T cell response [5]. A recent study showed that the CD28 costimulatory domain can recruit kinases like ZAP70 and PLC to enhance NK cell function [2], supporting our findings. Given the well-characterized role of CD28 in CAR signal transduction [2], we proceeded to investigate the downstream molecular changes by RNA-sequencing (Fig. 2C). The results revealed that 28z CAR-NK cells upregulated multiple genes in TLR, MAPK, and NF-κB signaling pathways compared to BBz CAR-NK cells (Fig. 2D, S4B), indicating that CD28 costimulation more effectively activates these pathways, boosting CAR-NK cell function.

## MAP3K8 is a key effector downstream of CD28 costimulation

Among the differentially expressed genes, MAP3K8, a kinase promotes inflammation through pathways such as MAPK signaling [3, 6], stood out (Fig. 2E). Given that 28z CAR-NK cells showed higher activation in pro-inflammatory pathway, we hypothesized MAP3K8 could mediate the functional differences between these CAR designs. We tested this hypothesis by inhibiting ZAP70 or targeting the downstream Akt pathway, which effectively abolished the differential MAP3K8 expression between 28z and BBz CAR-NK cells (Fig. S4C-D), suggesting that 28z CAR enhances MAP3K8 expression via the ZAP70-Akt signaling axis. In functional assays, knocking down MAP3K8 expression (Fig. S4E-F) or inhibiting its activity with a non-toxic dose of inhibitors (Fig. S4G) significantly reduced the cytotoxic activity of 28z CAR-NK92MI cells (Fig. 2F-I). Conversely, overexpressing MAP3K8 in BBz CAR-NK92MI cells improved their tumor-killing capacity and secretion of cytotoxic factors (Fig. 2J-L, S4H-I). MAP3K8 shows a similar function in CAR-YTS cells (Fig. S4J-O). Moreover, MAP3K8 also significantly enhanced the *in vivo* anti-tumor activity of BBz CAR-NK cells (Fig. 2M-Q). Mechanically, MAP3K8 overexpression activated the ERK1/2 pathway, and inhibition of this pathway nearly abolished the MAP3K8-mediated enhancement of CAR-NK cell function (Fig. S5). These results suggest that MAP3K8 is a critical effector downstream of the CD28 costimulatory signals, and targeting MAP3K8 could enhance BBz CAR-NK cell function.

In conclusion, 28z CAR-NK cells show superior anti-tumor activity by more efficiently recruiting ZAP70, reshaping the transcriptome, and upregulating molecules like MAP3K8. This highlights the benefits of CD28 costimulation in NK cells and offer insights and novel targets for optimizing CAR-NK therapies.

## Electronic supplementary material

Below is the link to the electronic supplementary material.

Supplementary Material 1

## Data Availability

No datasets were generated or analysed during the current study.

## Abbreviations

CAR: Chimeric antigen receptor
FBS: Fetal bovine serum
SP: Signal peptide
scFv: Single-chain variable fragment
H/T: Hinge/transmembrane domain
WT: Wild type
E: T ratio: Effector: target ratio
IP: Immunoprecipitation
RNA-Seq: RNA sequencing
LC-MS/MS: Liquid chromatography-tandem mass spectrometry
CBA: Cytometric bead array
IVIS: In vivo imaging systems
Tra: Trametinib
Mir: Mirdametinib
