# Supplementary material for: CD28 is superior to 4-1BB costimulation in generating CAR-NK cells for tumor immunotherapy
Source: Exp Hematol Oncol. 2025 Mar 3;14:28. doi: 10.1186/s40164-025-00618-7 (PMC11874623; doi:10.1186/s40164-025-00618-7)
Supplement: Supplementary file 1 — Supplementary Material 1 [file 40164_2025_618_MOESM1_ESM.docx]

**Supplemental figures and legends**


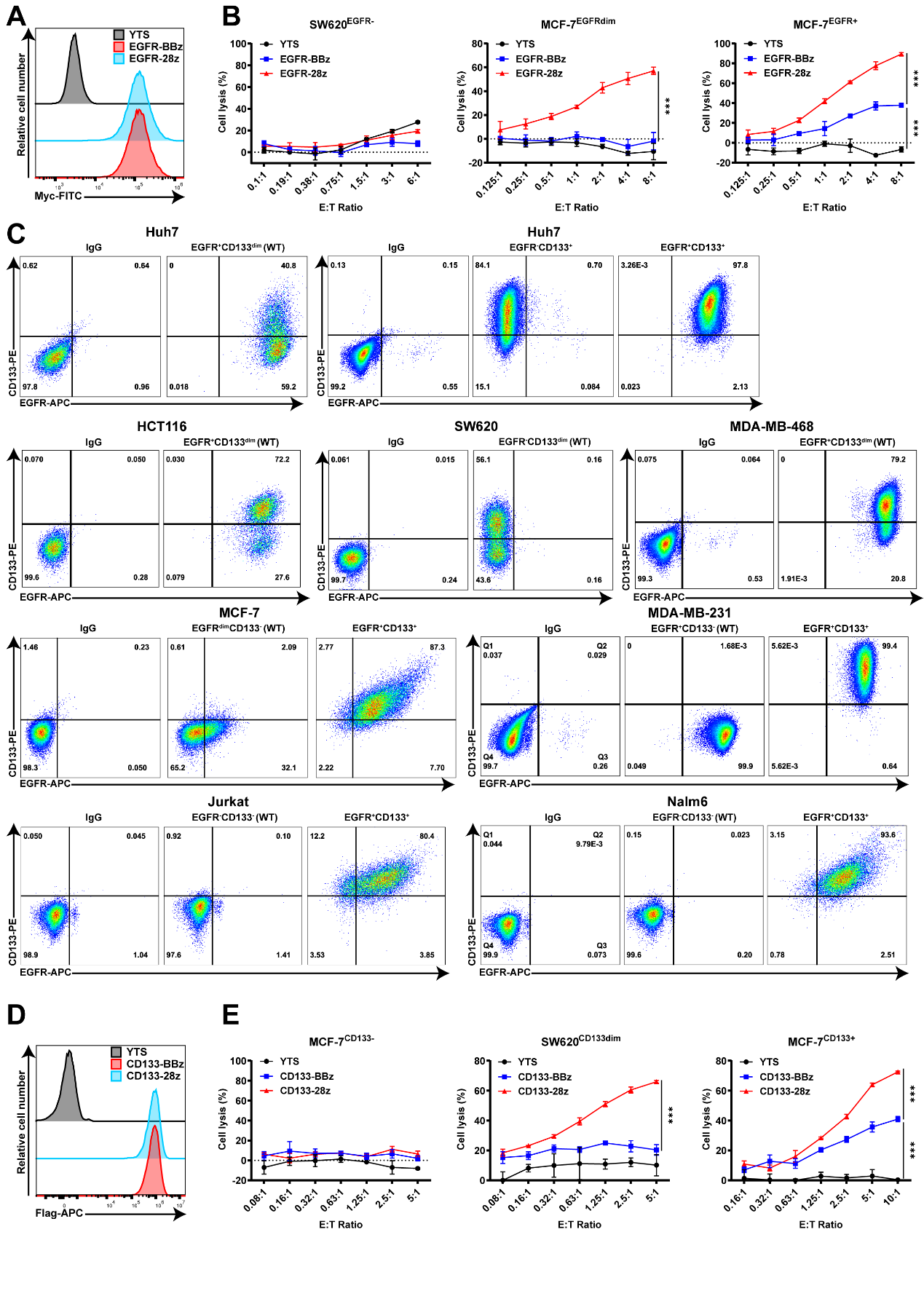


**Figure S1**. Cell construction and cytotoxicities of BBz and 28z CAR-YTS to target cells. (A) Flow cytometry analysis of EGFR-targeting CARs expression on the surface of YTS. (B) Cytotoxicities of EGFR-targeting CAR-YTS to antigen negative (left), low expression (middle), and positive (right) target cells. (C) EGFR and CD133 expression levels on target cells. WT represents antigens expression mode in wild-type target cells, and then target cells with multiple antigenic phenotypes were obtained by knocking out or overexpressing antigens. (D) Flow cytometry analysis of CD133-targeting CARs expression on the surface of YTS. (E) Cytotoxicities of CD133-targeting CAR-YTS to antigen negative (left), low expression (middle), and positive (right) target cells. All results are presented as mean ± SD. The differences were analyzed by two-way ANOVA analysis. ****p* < 0.001.


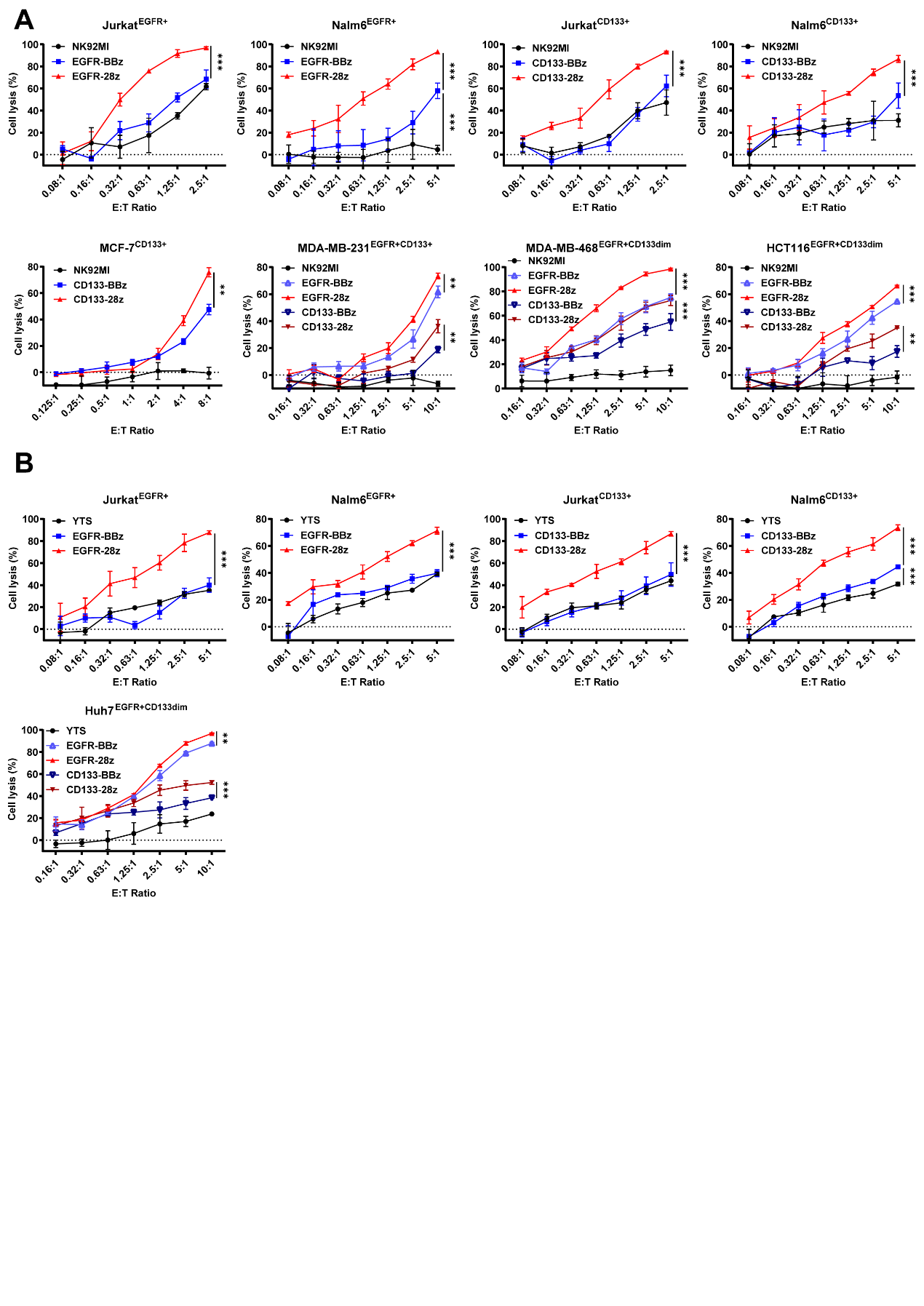


**Figure S2**. Cytotoxicities of BBz and 28z CAR-NK to target cells. In vitro cytotoxicities of CAR-NK92MI (A) and CAR-YTS (B) against antigen-positive hematologic and solid tumor cell lines. All results are presented as mean ± SD. The differences were analyzed by two-way ANOVA analysis. ***p* < 0.01; ****p* < 0.001.


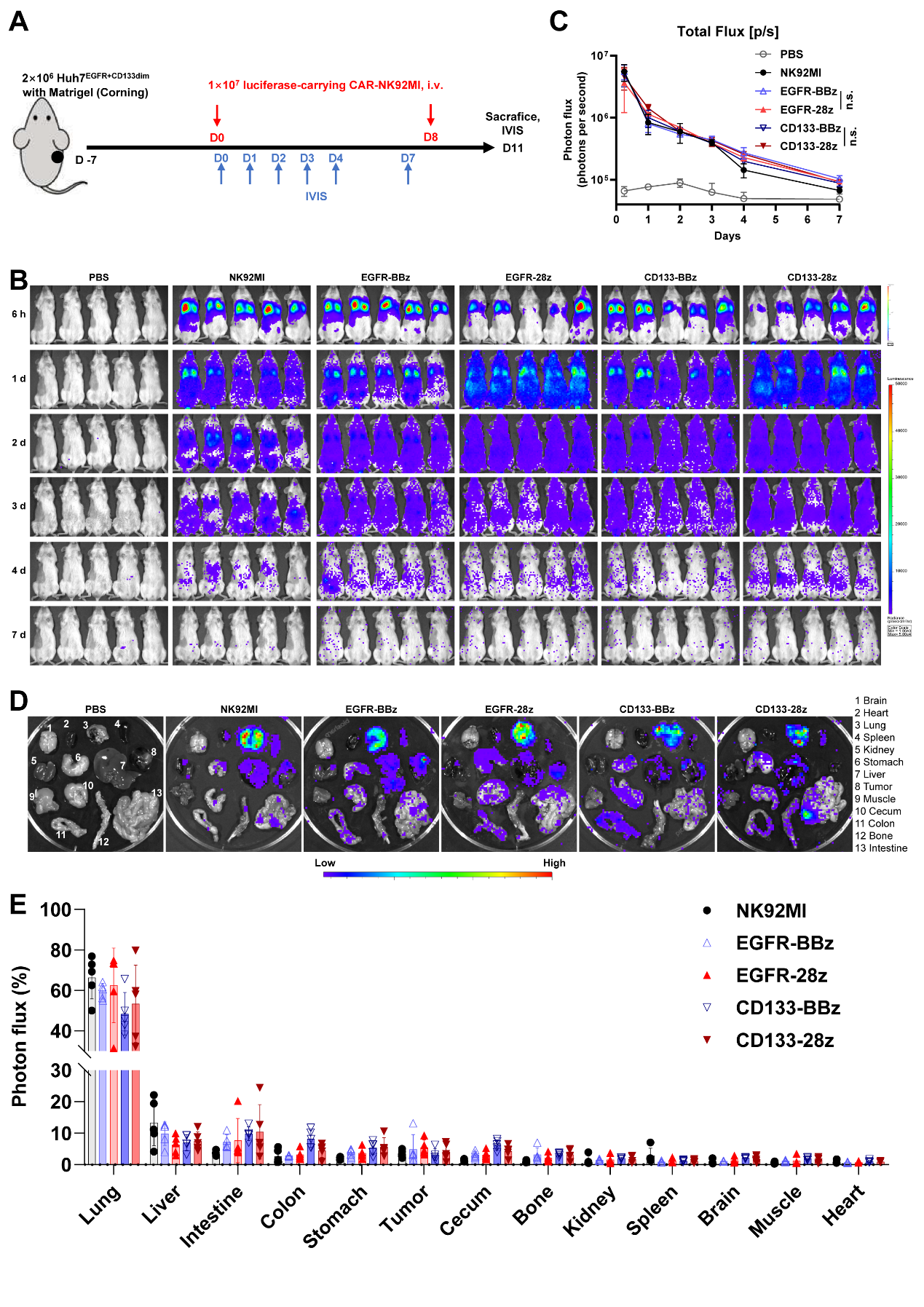


**Figure S3**. Persistence and distribution of CAR-NK92MI cells *in vivo*. (A) Diagram of experimental design of CAR-NK92MI Luc bio-distribution *in vivo*. Briefly, M-NSG mice were subcutaneously injected with 2×10^6^ Huh7 cells (day -7). On day 0, mice were randomly grouped (n = 5 mice per group) and injected (i.v.) with 1×10^7^ luciferase-carrying CAR-NK cells, and NK cell signals were detected by the *in vivo* imaging systems (IVIS) at the indicated time points. 20,000 U/mouse recombinant human IL-2 was injected (i.v.) one day after the cell infusion to improve the persistence of CAR-NK92MI *in vivo*. On day 8, mice received a second NK cell infusion and were dissected 3 days later to detect NK cell signals in various organs by IVIS. (B and C) Bioluminescence imaging (B) and quantification of the photons per second (C) were performed on the whole body at the indicated time points. (D) The mice were sacrificed on day11, and the NK cells in each dissected organs were detected by bioluminescence imaging. (E) Quantification of the photons per second as percentage of all organs. All results are presented as mean ± SD. The differences were analyzed by two-way ANOVA analysis. n.s., not significant.

**
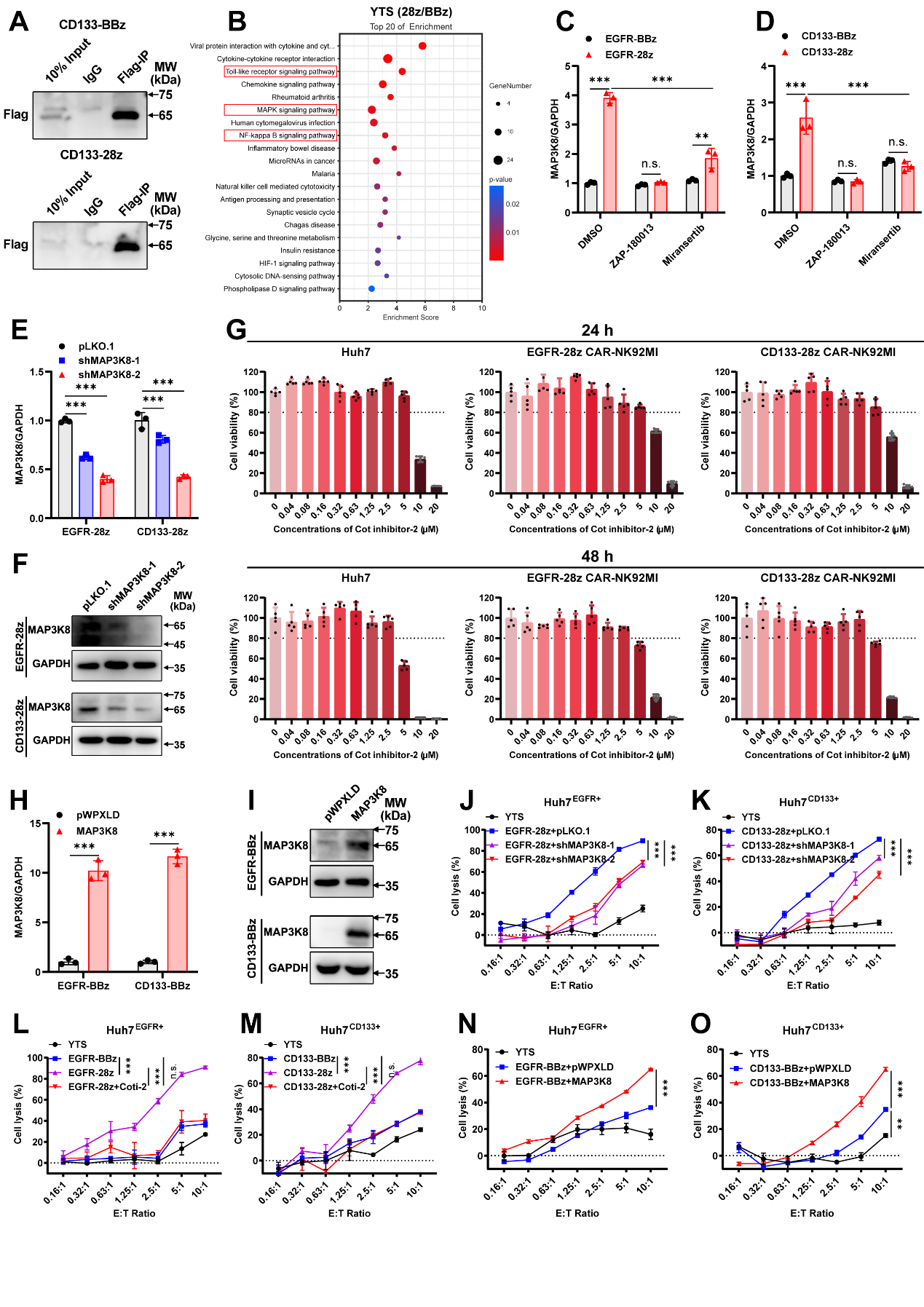
**

**Figure S4**. MAP3K8 affects the tumor killing function of CAR-NK cells. (A) Immunoblot analysis of Flag-CAR immunoprecipitation efficiency from CD133-targeting BBz and 28z CAR-NK92MI cells. (B) KEGG pathway enrichment bubble chart of 28z CAR-YTS versus BBz CAR-YTS. (C and D) Effect of ZAP70 and Akt inhibitors on MAP3K8 expression. EGFR (C) and CD133 (D)-targeting CAR-NK92MI cells were pretreated with ZAP70 inhibitor (ZAP-180013, 5 μM) or Akt inhibitor (Miransertib, 5 μM) for 1 h, and then cocultured with antigen positive Huh7 cells for 2 h in the presence of inhibitors. RNA extracted from magnetic beads sorted CAR-NK92MI cells were used for qPCR analysis. (E and F) The knockdown efficiency of MAP3K8 in 28z CAR-NK92MI was determined by qRT-PCR (E) and western blot (F). (G) Effect of Coti-2 on the cell viability of Huh7, EGFR-28z CAR-NK92MI, and CD133-28z CAR-NK92MI. (H and I) The overexpression efficiency of MAP3K8 in BBz CAR-NK92MI was determined by qRT-PCR (H) and western blot (I). (J-M) Effect of MAP3K8 knockdown (J, K) and inhibition (L, M) on cytotoxicity of 28z CAR-YTS to antigen positive Huh7 cells. For MAP3K8 inhibition, 28z CAR-YTS be pretreated with 2.5 μM Coti-2 for 1 h, then cocultured with target cells for 6 h in the presence of 2.5 μM Coti-2. (N and O) Effect of MAP3K8 overexpression on cytotoxicity of BBz CAR-YTS to antigen positive Huh7 cells. All results are presented as mean ± SD. The differences were analyzed by one-way or two-way ANOVA analysis. ***p* < 0.01; ****p* < 0.001; n.s., not significant.


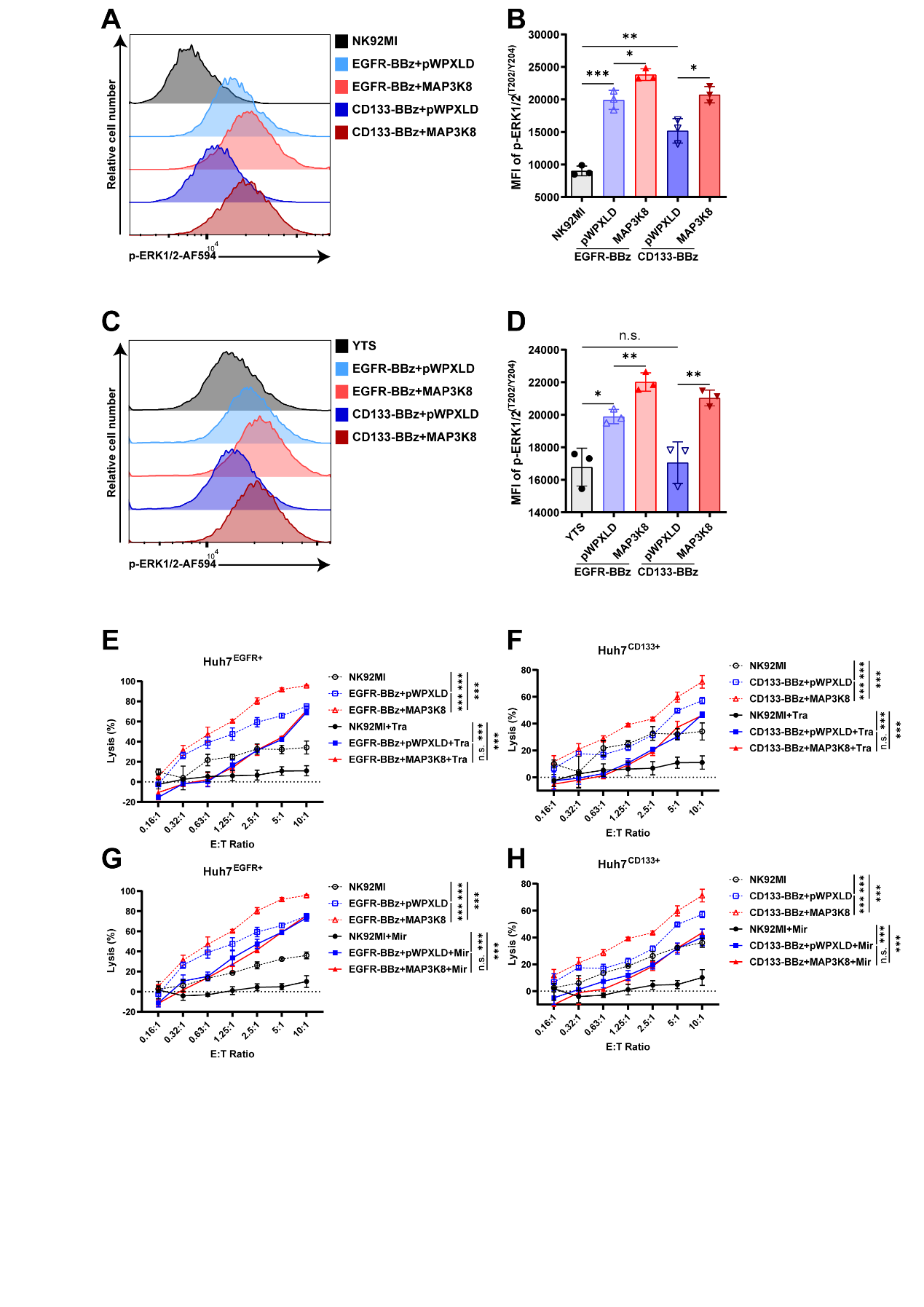


**Figure S5**. MAP3K8 affects the function of CAR-NK through the downstream MEK1/2-ERK1/2 pathway. (A-D) CAR-NK cells were cocultured with Huh7^EGFR+CD133+^ cells at the E:T ratio of 1:1 for 2 h, cells were then harvested for flow cytometry analysis of phosphorylated ERK1/2^(T202/Y204)^ levels in CD56^+^ cells. Flow cytometry (A) and MFI analysis (B) of p-ERK1/2 in CAR-NK92MI cells. Flow cytometry (C) and MFI analysis (D) of p-ERK1/2 in CAR-YTS cells. The differences for each group in (B) and (D) were analyzed by two-tailed unpaired Student’s *t* test. (E-H) Effects of MEK1/2 inhibitors Trametinib (Tra) and Mirdametinib (Mir) on the cytotoxicities of MAP3K8-overexpressed BBz CAR-NK92MI to target cells. EGFR-targeting (E) and CD133-targeting (F) BBz CAR-NK92MI were pretreated with 100 nM Tra for 1 h, then cocultured with antigen positive Huh7 cells for 6 h in the presence of 100 nM Tra and the cytotoxicities were detected. EGFR-targeting (G) and CD133-targeting (H) BBz CAR-NK92MI were pretreated with 1 μM Mir for 1 h, then cocultured with antigen positive Huh7 cells for 6 h in the presence of 1 μM Mir and the cytotoxicities were detected. The differences in (E-H) were analyzed by two-way ANOVA. All results are presented as mean ± SD. **p* < 0.05; ***p* < 0.01; ****p* < 0.001; n.s., not significant.

**Materials and methods**

**Cell lines and cell culture**

The human embryonic kidney cell line 293T, hepatocellular carcinoma cell line Huh7, breast adenocarcinoma cell line MCF-7, MDA-MB-231, MDA-MB-468, colon carcinoma cell line HCT116, and colon adenocarcinoma cell line SW620 were obtained from the Chinese Academy of Sciences cell bank (China) and cultured in DMEM medium (Gibco, USA) supplemented with 10 % FBS. The acute T cell leukemia cell line Jurkat, and acute lymphoblastic leukemia Nalm6 were obtained from the Chinese Academy of Sciences cell bank (China) and cultured in RPMI-1640 medium (Gibco, USA) supplemented with 10 % FBS. The IL-2 independent NK cell line NK92MI was obtained from Procell (China) and cultured with nucleosides-free minimum essential medium α (MEMα) (Gibco) supplemented with 12.5% horse serum (Gibco), 12.5% fetal bovine serum (FBS, Gibco), 0.1 mmol/L β-mercaptoethanol (Sigma-Aldrich, USA), 0.2 mmol/L inositol (Sigma-Aldrich), and 0.02 mM folic acid (Sigma-Aldrich). The clonal human NK cell line YTS was preserved in-house and grown in RPMI-1640 medium (Gibco) supplemented with 10% FBS. All cell lines were cultivated at 37˚C and 5% CO_2_ in a humidified incubator.

**Plasmid construction**

The full-length of human EGFR (NM_005228.5) and CD133 (NM_006017.3) expressing lentiviral plasmids (pLV3-CMV-EGFR and pLV3-CMV-CD133) were purchased from MiaoLing Plasmid Platform (China). Human EGFR knockout plasmids were constructed by subcloning the guide RNA-encoding sequences (gRNA-1: 5’-AAATCCTGCATGGCGCCGTG-3’ and gRNA-2: 5’-TGCTGACTATGTCCCGCCAC-3’) into the lentiCRISPRv2 lentiviral vector (#98290, Addgene, USA). Human MAP3K8 knockdown plasmids were constructed by subcloning the shRNA-encoding sequences (shMAP3K8-1: 5’- AGCCGCAGACCTACTAA-3’ and shMAP3K8-2: 5’-CTGACTTACAGGAATAT-3’) into the pLKO.1 lentiviral vector (#8453, Addgene, USA). The Luciferase and human MAP3K8 expressing plasmids were constructed by subcloning the coding sequences into the pWPXLD lentiviral vector (#12258, Addgene, USA). The full-length human MAP3K8 coding sequence was generated from NK92MI cells by RT-PCR. All CAR plasmids used pWPXLD as the vector and included the sequences that encoding the CD8α signal peptide (SP), the anti-EGFR or anti-CD133 single-chain variable fragment (scFv), Myc or Flag tag, the CD8α or CD28 hinge/transmembrane domain (H/T), the 4-1BB or CD28 costimulatory domain, and the CD3ζ signal domain. All sequence synthesis and sequencing were performed by Azenta Life Sciences (China).

**Lentiviral packaging and infection**

293T cells with 80% confluence in 10 cm dishes were used for lentiviral packaging. For every dish, co-transfecting lentiviral vector (10 μg) together with helper plasmids psPAX2 (7.5 μg) and pMD2.G (2.5 μg) into 293T cells. 48 h later after transfection, lentiviral-containing supernatant was collected and filtered through a 0.45 μm filter.

For target cell infection, 1×10^5^ Huh7, MCF-7, or SW620 cells were transduced using 2 mL lentiviral-containing supernatant with 10 μg/mL polybrene (Sigma-Aldrich). For NK cell infection, 10 mL lentiviral-containing supernatant was concentrated to 200 μL by 5×PEG8000 and then added to 2×10^5^ NK92MI or YTS cells, and a final concentration of 8 μg/mL polybrene was added to enhance infection efficiency. All positive cells were sorted by flow cytometry after expansion.

**Flow cytometry**

For cell surface antigen staining, 1×10^6^ cells were washed with 1 mL FACS buffer (PBS containing 1% FBS), and after centrifuge, the cell pellet was resuspended in 100 μL of FACS buffer diluted antibodies and incubated at 4°C for 20 min. The cells were then washed twice with FACS buffer and analyzed by flow cytometer (CytoFLEX, Beckman, USA). For phosphorylated ERK1/2^(T202/Y204)^ staining, FACS buffer washed cells were fixed and permeabilized, then resuspended in 100 μL of diluted phospho- ERK1/2^(T202/Y204)^ rabbit mAb and incubated at room temperature for 1 h. After twice wash with FACS buffer, the cells were resuspended in 100 μL of diluted Alexa Fluor® 594-conjugated goat anti-rabbit IgG antibody at room temperature for 1 h. The cells were then washed twice with FACS buffer and analyzed by flow cytometer. For cell sorting, the cells were prepared sterile and sorted by the flow cell sorter (MA900, SONY, Japan). FITC-conjugated anti-Myc antibody (clone SH1-26E7.1.3) was purchased from Miltenyi Biotech (Germany). APC-conjugated anti-DYKDDDDK (Flag tag) antibody (clone L5), APC-conjugated anti-human EGFR antibody (clone AY13), PE-conjugated anti-human CD133 antibody (clone 7), and PE-conjugated anti-human CD56 antibody (clone 5.1H11) were purchased from BioLegend (USA). Anti-phosphorylated ERK1/2^(T202/Y204)^ rabbit mAb was purchased from Cell Signaling Technology (USA). Alexa Fluor® 594-conjugated goat anti-rabbit IgG was purchased from Servicebio (China).

**In vitro cytotoxicity**

The in vitro cytotoxicity was performed using FFLuc reporter assay. In brief, 1×10^4^ cells/well luciferase-expressing target cells adhered to the 96-well black plate, and the CAR-NK cells were added according to the indicated effector: target (E: T) ratios and cocultured for 6 h. The medium in each well was then replaced with 150 μg/mL D-Luciferin potassium salt (Beyotime, China) and incubated at 37°C for 5 min. The signals were measured by the Omega multifunctional microplate reader (BMG LABTECH, Germany). The cell-free wells were set as the blank, and the wells with only target cells were set as the negative control. The mean value of the blank was subtracted from all wells and then the in vitro cytotoxicity was calculated by the following formula: (1-sample/negative control)×100%.

**Cytokine release assay**

CAR-NK cells were cocultured with target cells in 96-well plates at an E: T ratio of 2:1 for 24 h. The supernatant from the co-culture system was collected and the secretion of cytokines was detected using the LEGENDplex™ Human CD8/NK Panel (BioLegend) according to the manufacturer’s instructions.

**Animal studies**

All animal procedures were approved by the institutional animal care and use committee of Shenzhen Institutes of Advanced Technology, Chinese Academy of Sciences. 8 weeks old male NOD.Cg-*Prkdc^scid^Il2rg^em1Smoc^* (M-NSG) mice were purchased from the Shanghai Model Organism Center, Inc. (China) and housed in the Shenzhen Institutes of Advanced Technology animal facility under pathogen-free conditions. 5×10^6^ Huh7 cells in 100 μL of PBS supplement with 25% Matrigel® Matrix (Corning, USA) were subcutaneously injected into the right flank. When tumor nodes were palpable (approximately 100 mm^3^), the mice were randomly divided into three groups and received the 1×10^7^ Mock or CAR-NK92MI cells in 200 μL PBS intravenously (i.v.). All mice were injected (i.v.) with recombinant human IL-2 (20,000 U/mouse, T&L Biotechnology, China) in the next day. The body weight and tumor size were collected every day. The tumor volume was calculated by the formula: Tumor volume (mm^3^) = ½(Length×Width^2^).

**Bioluminescence Imaging**

*In vivo* imaging systems (IVIS) was used for assessing Luc bio-distribution in vivo, mice were injected intraperitoneally (i.p.) with 200 μL D-Luciferin potassium salt (Beyotime, China, 15 mg/mL) in sterile PBS. After 10 mins, the bioluminescence signals of the whole body or dissected organs of the mice were detected with Caliper Spectrum IVIS (PerkinElmer) and data analyzed using the Living Image software (PerkinElmer, USA). The signal was quantified in photons/second.

**Protein extraction and Western blot**

Cells were collected and washed with PBS, the cell pellets were lysed in RIPA buffer supplemented with protease inhibitor and phosphatase inhibitor (Beyotime). After denaturation, protein samples were separated by SDS-PAGE, transferred to a PVDF membrane, and blotted with specific antibodies. Proteins in the membrane were visualized by an enhanced Chemiluminescense Detection Kit (Millipore, USA). The antibodies against the following antigens were used: anti-Cot (clone H-7, Santa Cruz, USA), HRP-conjugated anti-GAPDH (clone 1A6, Bioworld, USA), HRP-conjugated anti-Flag (clone 29E4.G7, Rockland, USA), HRP-conjugated goat anti-mouse IgG (SeraCare, USA).

**Immunoprecipitation and LC-MS/MS protein identification**

After CAR-NK were co-cultured with Huh7 for 1 h, the cells were harvested and washed with PBS. The cells were then lysed in immunoprecipitation (IP) buffer (150 mM NaCl, 50 mM Tris-HCl, pH 7.4, 50 mM EDTA, 1.0% NP-40, 1 mM PMSF). IP was performed using Protein A/G Magnetic Beads (MCE, USA). In brief, anti-Flag antibody-conjugated magnetic beads or Ig control-conjugated magnetic meads were incubated with 500 μL cell lysate overnight at 4°C with rotation. The captured immunoprecipitates were washed five times with IP buffer and boiled in 2×loading buffer. The eluted proteins were detected by Western blot analysis.

For LC-MS/MS protein identification, the washed magnetic beads were stored at -80°C and handed over to Luming Bio (China) for LC-MS/MS analysis.

**RNA isolation and qRT-PCR**

Total RNA was extracted using TRIzol reagent (Invitrogen, USA) and reversed into cDNA using FastKing One Step RT-PCR Kit (TIANGEN, China). Quantitative Real-Time PCR (qRT-PCR) was performed with Hieff® qPCR SYBR Green Master Mix (Yeasen, China) on CFX96 Real-Time System (Bio-Rad, USA). The primers were synthesized by Azenta Life Sciences and the sequences are as follows: MAP3K8 (F: 5’-CTCCCCAAAATGGACGTTACC-3’ and R: 5’-GGATTTCCACATCAGATGGCTTA-3’), GAPDH (F: 5’-CTGGGCTACACTGAGCACC-3’ and R: 5’-AAGTGGTCGTTGAGGGCAATG-3’).

**RNA-Seq**

CAR-NK and Huh7 were co-cultured at an E: T ratio of 1:1 for 2 h, and then the cells were harvested and sorted with MojoSort™ Human CD56 Nanobeads (BioLegend) to obtain CD56 positive cells, which were lysed with TRIzol reagent and subjected to RNA sequencing by Beijing Genomics Institute (BGI Genomics Co., Ltd, China). Dr. TOM, an in-house customized data mining system of the BGI was used for further bioinformatics analysis.

**Cell viability analysis**

The effect of Cot inhibitor-2 (Coti-2, MCE) on the viability of Huh7 and CAR-NK92MI cells was measured by the MTS kit (Promega, USA) according to the protocol provided by the manufacturer. Briefly, cells were seeded in 96-well plates at a density of 1×10^4^ cells/well in 100 μL medium, various concentrations of Coti-2 were added and culture the cells at the incubator for 24 h or 48 h. Subsequently, 20 μL MTS/PMS mixture solution was added to each well and the plates were incubated at 37°C for 1-4 h. The optical density of each well was determined at 490 nm with a microplate reader (Multiskan FC, Thermo Scientific, USA).

**Statistical analysis**

All analyses were performed using GraphPad Prism 8.3.0 software. Data were presented as mean±SD. Two-tailed unpaired Student’s *t* test and ANOVA analysis were used to analyze differences for the data. *p*<0.05 (*), *p*<0.01(**), and *p*<0.001(***) were considered statistically significant, n.s. indicates no significant difference.
